# *In vivo* N-Terminomics Highlights Novel Functions of ADAMTS2 and ADAMTS14 in Skin Collagen Matrix Building

**DOI:** 10.3389/fmolb.2021.643178

**Published:** 2021-03-19

**Authors:** Cédric Leduc, Laura Dupont, Loïc Joannes, Christine Monseur, Dominique Baiwir, Gabriel Mazzucchelli, Christophe Deroanne, Alain Colige, Mourad Bekhouche

**Affiliations:** ^1^ Laboratory of Connective Tissues Biology, GIGA-Cancer, University of Liège, Liège, Belgium; ^2^ GIGA Proteomic Facility, GIGA–Interdisciplinary Cluster for Applied Genoproteomics, University of Liège, Liège, Belgium; ^3^ Tissue Biology and Therapeutic Engineering, Centre National de la Recherche Scientifique/University of Lyon Unité Mixte de Recherche 5305, Lyon, France; ^4^ Faculté d’Odontologie de Lyon, Université de Lyon, Université Lyon 1, Lyon, France

**Keywords:** ADAMTS, collagen, Ehlers-Danlos Syndrome (EDS), degradomics, TAILS, N-Terminomics

## Abstract

A disintegrin and metalloproteinase with thrombospondin type I motif (ADAMTS)2 and ADAMTS14 were originally known for their ability to cleave the aminopropeptides of fibrillar collagens. Previous work using N-terminomic approach (N-TAILS) *in vitro* led to the identification of new substrates, including some molecules involved in TGF-β signaling. Here, N-TAILS was used to investigate the substrates of these two enzymes *in vivo*, by comparing the N-terminomes of the skin of wild type mice, mice deficient in ADAMTS2, in ADAMTS14 and in both ADAMTS2 and ADAMTS14. This study identified 68 potential extracellular and cell surface proteins, with the majority of them being cleaved by both enzymes. These analyses comfort their role in collagen matrix organization and suggest their implication in inflammatory processes. Regarding fibrillar collagen, this study demonstrates that both ADAMTS2 and ADAMTS14 are involved in the processing of the aminopropeptide of alpha1 and alpha2 type V collagen. It also revealed the existence of several cleavage sites in the Col1 domain and in the C-propeptide of type I collagens. In addition to collagens and other extracellular proteins, two major components of the cell cytoskeleton, actin and vimentin, were also identified as potential substrates. The latter data were confirmed *in vitro* using purified enzymes and could potentially indicate other functions for ADAMTS2 and 14. This original investigation of mouse skin degradomes by N-terminomic highlights the essential role of ADAMTS2 and ADAMTS14 in collagen matrix synthesis and turnover, and gives clues to better understand their functions in skin pathophysiology. Data are available via ProteomeXchange with identifier PXD022179.

## Introduction

Ehlers-Danlos syndrome (EDS) encompasses a group of inherited diseases caused by mutations affecting genes involved in the homeostasis of connective tissues (Beighton et al., 1998). A significant number of these genes are directly related to the biology of fibrillar collagens. Mutations in type V and type III procollagens are the main causes of, respectively, the “classical” and the “vascular” types of EDS. Missense mutations in type I procollagens can also lead to rare forms of classical and vascular EDS, while complete or partial skipping of exon six in alpha1 type I (COL1A1) or alpha2 type I (COL1A2) is responsible for arthrochalasia EDS, a condition mainly characterized by congenital bilateral hip dislocation, severe generalized joint hypermobility with multiple dislocations/subluxations and skin hyperextensibility (De Paepe and Malfait, 2012; Malfait and De Paepe, 2014). Electron microscopy of skin specimens shows loosely and randomly organized collagen fibrils with a smaller and variable diameter, and an irregular outline (Colige et al., 2004). The molecular basis behind this clinical picture is the absence of aminopropeptide cleavage of the mutated chain (either alpha1 or alpha2) since exon six encodes the cleavage site cleaved by aminoprocollagen peptidases (ADAMTS2, 3 and 14) (Colige et al., 1999). Null mutations in ADAMTS2, the main aminoprocollagen peptidase, lead also to EDS, but, surprisingly, not to the arthrochalasia EDS as reported for its mutated substrates. Instead, absence of ADAMTS2 activity causes dermatosparaxis EDS presenting with extreme skin fragility, characteristic craniofacial features, redundant skin with excessive skin folds at the wrists and ankles, umbilical hernia and severe bruising with a risk of subcutaneous hematomas and hemorrhages (Colige et al., 1999). In these patients and in animal models of deficiency in Adamts2 (TS2^−/−^) electron microscopy shows collagen fibrils with a highly typical hieroglyphic or ribbon-like pattern. These differences in the morphology of collagen fib rils in arthrochalasia and dermatosparaxis, while persistence of N-propeptides is observed in both diseases, suggested that ADAMTS2 has possibly substrates other than type I procollagens.

Because of their close similarities with ADAMTS2, the consequences of mutations affecting ADAMTS3 and ADAMTS14 have been evaluated in mouse models (Janssen et al., 2016; Dupont et al., 2018). ADAMTS3 has been shown to be required for the formation of lymphatic network by its capacity to cleave pro-VEGF-C into fully active VEGF-C able to bind its receptors (Janssen et al., 2016). Regarding ADAMTS14, KO-mice do not display obvious specific phenotype despite the fact that biallelic null mutations have been predicted to be pathogenic using dedicated browser such as “GnomAD”. In the same line, mice deficient for both Adamts2 and Adamts14 (TS2^−/−^TS14^−/−^) display an aggravated phenotype as compared to TS2^−/−^ mice, again indicating further roles and substrates for ADAMTS14 (Dupont et al., 2018). In order to have first indications about the diversity of the substrates of ADAMTS2, 3 and 14, a proteomic analysis dedicated to the identification of protease substrates (N-TAILS) was performed in cell culture models (Bekhouche et al., 2016). This study showed that the repertoire of substrates of these “so-called” aminoprocollagen peptidases extends beyond fibrillary procollagens. Although most useful because of their relatively low complexity, these *in vitro* models do not recapitulate the *in vivo* situation. As an example, fibrillary procollagens are secreted in the culture medium as uncleaved precursors, whereas in connective tissues *in vivo* they stay concentrated close to the producing cells and are fully matured.

In order to clarify why arthrochalasia and dermatosparaxis EDS display different clinical phenotypes (Van Damme et al., 2016) and to investigate the diverse functions of ADAMTS2 and ADAMTS14 *in vivo* (Dupont et al., 2018), N-Tails analysis has been performed on skin samples from wild-type mice (Wt) and TS2^−/−^, TS14^−/−^ and TS2^−/−^TS14^−/−^.

## Materials and Methods

### Reagents

The hyperbranched polyglycerol-aldehyde polymer was purchased from Flintbox (University of British Columbia, Vancouver, BC, Canada). Isobaric tag for relative and absolute quantitation (iTRAQ) labels are from AB Sciex (Concord, ON, Canada). Porcine trypsin was purchased from Promega (#V511A; Madison, WI, United States). Ultrafiltration devices were purchased from Merck Millipore. All other reagents were purchased from Sigma-Aldrich (Saint-Quentin Fallavier, France).

### Cell Lines

Fibroblasts were obtained from the dermis of healthy donors (normal fibroblasts, NF) and of a patient suffering from the dermatosparactic type of the Ehlers-Danlos syndrome (dermatosparactic fibroblasts, DF) (Nusgens et al., 1992). Cells were cultured in DMEM (Lonza) supplemented with 10% fetal bovine serum (FBS, Lonza). For collagen production, cells were cultured in DMEM supplemented with 1% FBS and 50 μg/ml 2-phospho-l-ascorbic acid (Sigma Cat#49752). The conditioned medium was collected after 48h, centrifuged for 15 min at 4,000 rpm at 4°C. The supernatant was conserved at −80°C before collagen purification.

### Skin Proteome Preparation

The experiment was conducted in triplicate using twelve 8-week-old adult mice (2 males and one female of each genotype (Wt, TS2^−/−^, TS14^−/−^ and TS2^−/−^/TS14^−/−^)). The investigation on mice was reviewed and approved by Ethics Committee for Animal Use and Care of the University of Liege (Belgium) (protocol N°1,109). A square (1 cm^2^) of shaved skin was collected from the anteroventral part of the mice and incubated 1 h at 4°C under shaking in 3 ml of 50 mM HEPES sodium salt (pH 7.5), 2 mM CaCl_2_ and 150 mM NaCl containing a cocktail of inhibitors of proteases and phosphatases (MS-SAFE, Sigma). Skin pieces were harvested and crushed with ceramic beads (MagNA Lyser Green Beads, Roche, n°03358,941,001) using the MagNA Lyser Instrument (Roche) in 90% (V/V) tissue protein extraction reagent (Thermo Scientific #78510), 10% (V/V) HEPES sodium salt (pH 7.5), 2 mM CaCl_2_, 150 mM NaCl containing the MS-SAFE inhibitors cocktail. Crushed samples were centrifuged 5 min at 8,000 rpm at 4°C and the protein concentrations estimated (Bradford assay (Bradford, 1976)) in the collected supernatants and adjusted to 1.0 mg/ml. Samples of 500 µg of proteins for each condition were used for iTRAQ-TAILS labeling (Kleifeld et al., 2011; Bekhouche et al., 2016). Proteomic analyses were performed at the GIGA Proteomic platform on the ESI-Q Exactive (ThermoFisher) coupled with a 2D-RP/RP liquid chromatography (2D-RP/RP NanoAcquity UPLC, Waters, Milford, United States) for the peptide fractionation in three fractions.

### Proteomic Data Analysis

Proteomic data analysis was previously described (Bekhouche et al., 2016). Briefly, in a first step, peptides were identified using Mascot (version 2.2.06; Matrix Science Inc., Boston, MA, United States) and allowing non-tryptic cleavages and two missed cleavages/peptide. Carbamidomethyl cystein was set as a fixed modification, and other modifications were set as variable: N-terminal acetyl, deamidation (NQ), Pyro-glu (N-term E), Pyro-Gln (N-term Q), Oxidation (M), iTRAQ (K), iTRAQ (Y) and iTRAQ (N-term). Peptide tolerance was set at 0.02 Da.

The tandem mass spectrometry (MS/MS) data were analyzed using the TransProteomicPipeline (TPP). The PeptideProphet and ProteinProphet software programs, embedded into TPP, were used to validate protein and peptide assignment. The nontryptic model was omitted in the PeptideProphet parameters. The error rate to validate proteins or peptides was respectively set at 2 and 5%. Then, Clipper software was used to determine the upper and lower cutoffs corresponding to 3-sigma calculated from the normal distribution of the log2(P:C ratio) from natural mature N termini. A Gaussian error function was used to calculate a *p* value that reflects the probability of a peptide to be a false-positive. A peptide with a P:C ratio above or below the 3-sigma cutoff has 99,8% chance to be dependent of the studied protease (auf dem Keller and M Overall, 2012). The cutoffs for each experiment are reported in Supplementary Table S4. The mass spectrometry proteomics data have been deposited to the ProteomeXchange Consortium via the PRIDE (Perez-Riverol et al., 2019) partner repository with the dataset identifier PXD022179 and 10.6019/PXD022179.

### Biological Process Analysis

The biological processes have been investigated using the Panther database (PANTHER version 14: more genomes, a new PANTHER GO-slim and improvements in enrichment analysis tools (Mi et al., 2019)). Statistical overrepresentation tests were performed using the whole *Mus musculus* genome as reference dataset. The *p*-Values are determined using the Fisher’s Exact test corrected by the determination of the false discovery rate (fdr). The fdr was below 0.05 for all the reported biological process.

The Venn diagram were drawn using the BioVenn software (BioVenn—a web application for the comparison and visualization of biological lists using area-proportional Venn diagrams (Hulsen et al., 2008)).

### Purification of Recombinant ADAMTS2 and 14

Recombinant ADAMTS2, its inactive mutant, or ADAMTS14 were produced in HEK293 cells, purified and quantified as previously described (Colige et al., 2005). Briefly, recombinant proteases were purified, using Concanavalin A-Sepharose and Heparin-Sepharose columns, from 1 L of serum-free medium conditioned during 48 h. Proteases were recovered in 50 mM Tris, pH 7.5, 1 M NaCl, 2 mM CaCl_2_.

### Analysis of Fibrillar Collagens Degradation

Collagen from dermatosparactic or normal calf skin was extracted and purified according to a reported procedure (Nusgens and Lapiere, 1979; Colige et al., 1995). Collagen from fibroblasts cultures, was concentrated by adding ethanol to the conditioned medium (see above, 2.2) at a final concentration of 33% (V/V). After overnight incubation at 4°C, precipitated collagen was recovered by centrifugation (7,000 rpm, 40 min, 4°C). Pellets were solubilized in 0.1 M acetic acid (pH 2.9) under shaking at 4°C for 18 h. Non-solubilized contaminants were excluded by centrifugation (17,000 rpm, 40 min, 4°C) and the supernatant containing the collagen was neutralized by adding 1 M Tris base solution (at 1/10 of the final volume). Collagen cleavage was analyzed by incubation of 10 µg of type I collagen with recombinant ADAMTS2 or 14 (200 nM for 16 h at 37°C) in 50 mM Tris (pH 7.5), 0.5 M NaCl, 2 mM CaCl_2_, in the presence or absence of 25 mM EDTA used as inhibitor of ADAMTS metalloproteinase activity. Collagens were used in native form or after thermal denaturation at 95°C for 10 min. Digestion products were separated by SDS-PAGE and stained with Coomassie Blue.

### Analysis of Cleavage of Intracellular Substrates

Cell lysates from human normal fibroblasts (NF) were prepared by sonication and incubated with recombinant human ADAMTS2 and/or 14 (200 nM for 16 h at 37°C) in 50 mM Tris (pH 7.5), 0.5 M NaCl, 2 mM CaCl_2_, in the presence or absence of 25 mM EDTA. Actin and vimentin cleavages were analyzed by Western blot using polyclonal anti-alpha-actin-1 (A2066, Sigma) and polyclonal anti-vimentin (VI008–01, Quartett).

### Determination of Cleavage Site Specificity

Amino acid sequence logos, corrected by the natural abundance of amino acids in the human proteome, were generated using the iceLogo software package (Colaert et al., 2009). Analyses were based on the cleavage sites determined by proteomics for all the potential extracellular substrates, without type I collagen cleavage sites or without all fibrillar collagens.

## Results

### N-Terminomic Analysis of ADAMTS2 and ADAMTS14 Skin Degradome

**FIGURE 1:**
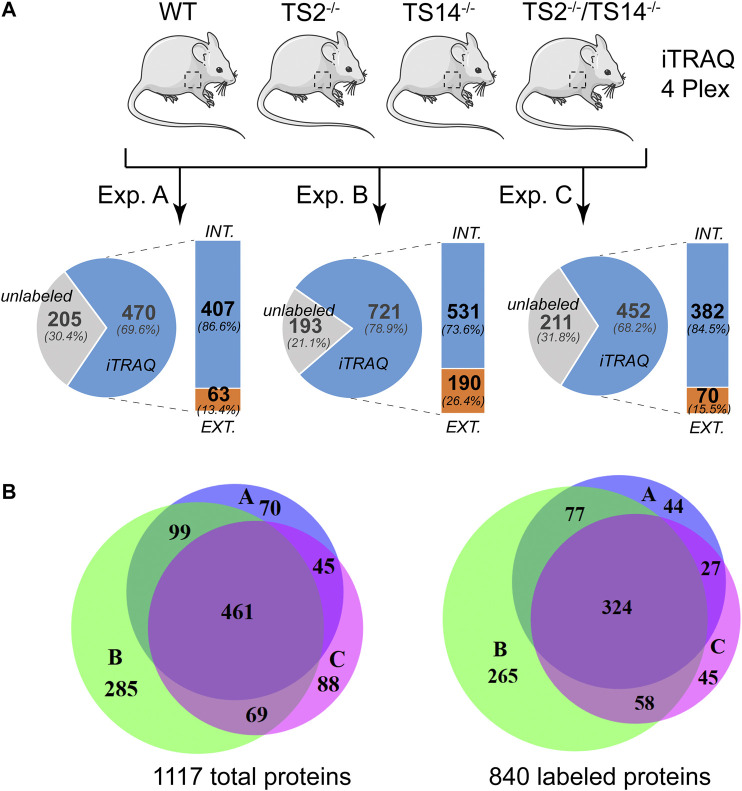
N-terminomic analysis of ADAMTS2 and 14 in mouse skin. **(A)** Schematic view of the experimental method to assess the potential substrates of ADAMTS2 and/or ADAMTS14 in mouse skin and to investigate their biological function *in vivo*. Three independent iTRAQ-TAILS analyses using skin proteome of each genotypes (4-plex) have been performed. As a general procedure, a protease/control (P/C) ratio above or below the three sigma cutoff from the normal distribution of natural N-termini is considered to be related to the studied protease. Here, the ratio Wt/TS2^−/−^ and TS14^−/−^/TS2^−/−^TS14^−/−^ were used to investigate the ADAMTS2 substrates, the ratios Wt/TS14^−/−^ and TS2^−/−^/TS2^−/−^TS14^−/−^ were used to investigate the ADAMTS14 substrates and the ratio Wt/TS2^−/−^TS14^−/−^ was used to investigate the substrates common to ADAMTS2 and ADAMTS14. **(B)** Venn diagram showing the number of specific or common total **(left)** or labeled **(right)** proteins identified in each experiment. Experiment A is in blue, B in green and C in purple. Venn diagrams were generated using the online BioVenn software (Hulsen et al., 2008).

ADAMTS2 and ADAMTS14 are clearly implicated in several physio-pathological processes (Colige et al., 1999; Kesteloot et al., 2007; Dubail et al., 2010; Dupont et al., 2018; Wang et al., 2020). To better understand their *in vivo* roles in skin physiology and extracellular matrix homeostasis, N-terminomic experiments have been performed on skin of mice, either wild type (Wt), deficient in Adamts2 (TS2^−/−^), deficient in Adamts14 (TS14^−/−^) and deficient in both Adamts2 and Adamts14 (TS2^−/−^TS14^−/−^) (Figure 1A). For each experiment, proteins extracted from the four genotypes were labeled with specific iTRAQ labels for quantification by mass spectrometry (MS/MS). This 4-plex experiment was performed in triplicate. The proteomic analysis shows that between 68 and 79% of the proteins were labeled and have therefore been used for relative quantification. Among the labeled proteins between 13 and 26% are extracellular. By using Venn diagrams as analytical tool, a total of 1,117 proteins, including 840 with an iTRAQ labeled peptide, were identified in the three experiments, but only 41 and 38% of them were common to the three experiments, for unlabeled and labeled proteins, respectively (Figure 1B). As actual substrates might have been missed during the proteomic analyses, especially when their abundance is low, the data of these three experiments can be considered as complementary. In order to take this limitation into account but to generate also high confidence data, a labeled peptide was considered to identify a candidate substrate when it was observed in at least two experiments with a similar Protease/Control (P/C) fold change (either increased or decreased).

**FIGURE 2:**
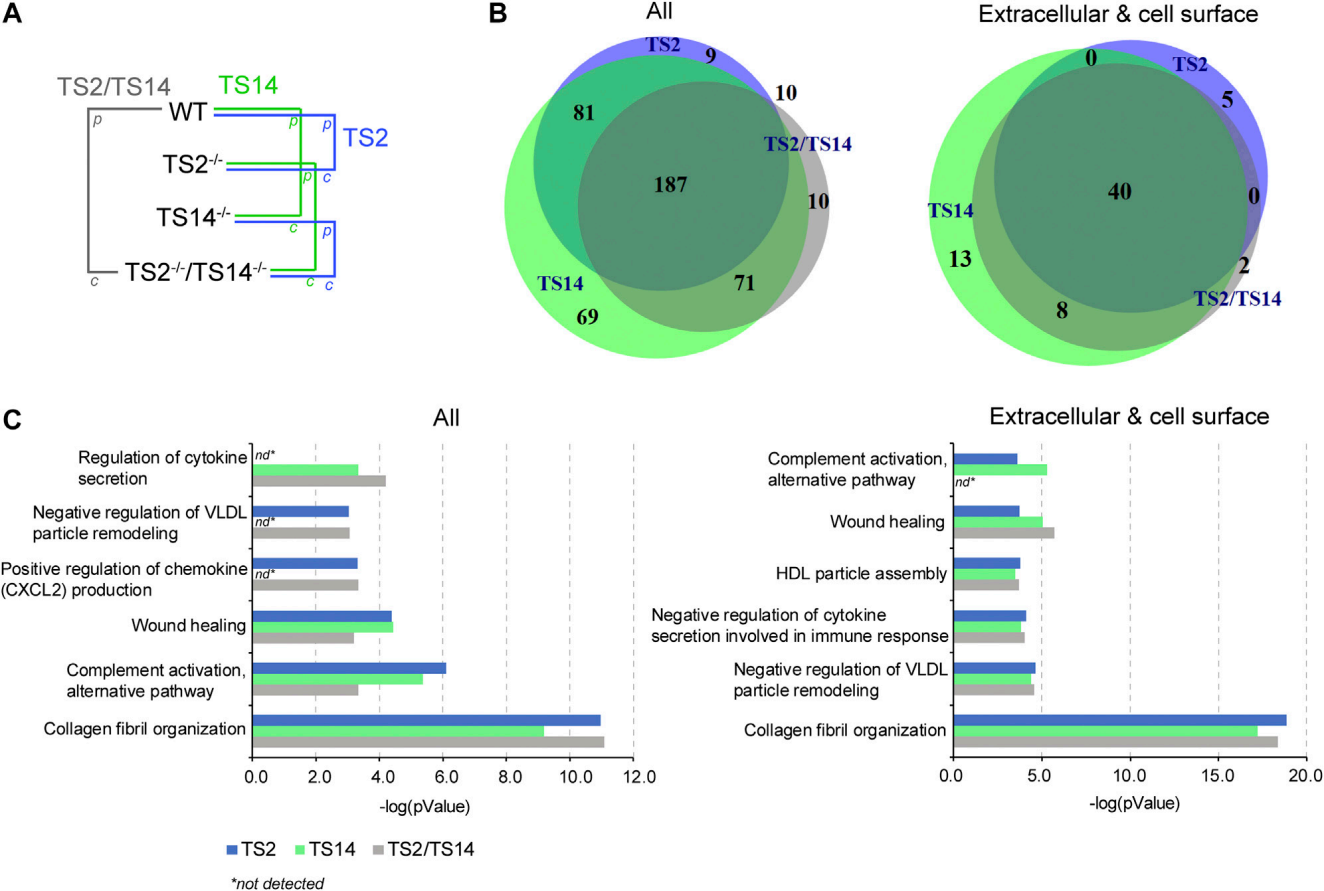
ADAMTS2 and 14 degradomes in mouse skin and their biological processes. **(A)** Schematic view of the multiple comparisons of the P/C ratios analyzed for each iTRAQ-TAILS experiment (n = 3). The function of the ADAMTS2 (in blue) is investigated by the comparison of wild type (Wt) mice with those deficient for ADAMTS2 (TS2^−/−^) and from the comparison of the mice deficient for ADAMTS14 (TS14^−/−^) with those deficient for ADAMTS2 and ADAMTS14 (TS2^−/−^TS14^−/−^). The potential substrates of ADAMTS14 (in green) were identified from the comparison of Wt mice with the mice deficient for ADAMTS14 (TS14^−/−^) and from the comparison of the mice deficient for ADAMTS2 (TS2^−/−^) with those deficient for ADAMTS2 and ADAMTS14 (TS2^−/−^TS14^−/−^). The common substrates of ADAMTS2 and 14 (red) were obtained from the comparison between Wt mice and TS2^−/−^TS14^−/−^ mice, and by combining data obtained specifically for Adamts2 and for Adamts14. **(B)** Venn diagram showing the common and specific N-terminally labeled proteins related to ADAMTS2 (blue), ADAMTS14 (green) or ADAMTS2 and ADAMTS14 (green). All the 437 unique proteins (287 identified by comparing Wt to TS2^−/−^, 408 by comparing Wt to TS14^−/−^ and 278 by comparing Wt to TS2^−/−^TS14^−/−^) showing a P/C ratio above or below three sigma cutoff from the normal distribution of natural N-termini are reported on the left. The 68 extracellular and cell surface proteins related to ADAMTS2 or ADAMTS14 activities (45 by comparing Wt to TS2^−/−^, 61 by comparing Wt to TS14^−/−^ and 50 by comparing Wt to TS2^−/−^TS14^−/−^) are reported on the right. **(C)** Biological processes related to all the proteins affected by ADAMTS2 and ADAMTS14 in mouse skin **(left panel)** or related to the extracellular and cell surface substrates **(right panel)**. The biological processes have been identified using the Panther database (Mi et al., 2019). Statistical Overrepresentation test was performed using the whole *Mus musculus* as reference dataset, the pValue is determined using the Fisher’s Exact test and corrected by the determination of the false discovery rate (fdr). The fdr is below 0.05 for all the biological processes reported. nd: not detected.

The degradomes (proteolytic events related to a proteolytic enzyme) are determined from the Protease/Control (P/C) ratios significantly different from the normal distribution of the natural N-termini. They include the proteins cleaved directly by the studied protease and also indirectly by the activation of other proteases or regulatory pathways. In this study, any N-terminally iTRAQ labeled peptide was considered to be part of the ADAMTS2 and/or ADAMTS14 degradomes when its P/C ratio was above or below a 3-sigma cut-off from the normal distribution of natural N-termini, including those obtained after the removal of the signal peptide. The degradome of ADAMTS2 has been determined from Wt vs TS2^−/−^ and from TS14^−/−^ vs TS2^−/−^TS14^−/−^ ratios, and that of ADAMTS14 has been determined from Wt vs TS14^−/−^ and from TS2^−/−^ vs TS2^−/−^TS14^−/−^ ratios, while that of ADAMTS2 and ADAMTS14 together has been determined from Wt vs TS2^−/−^TS14^−/−^ ratios (Figure 2A). In this analysis, the whole degradome of ADAMTS2 and/or ADAMTS14 was found to be composed of 437 proteins, including 68 proteins secreted or anchored at the cell surface. Forty extracellular and cell surface proteins were related to both ADAMTS2 and ADAMTS14 activities, while five are specific of ADAMTS2 and 21 (13 + 8) of ADAMTS14. Of note, two proteins were detected only when comparing Wt and TS2^−/−^TS14^−/−^, the coagulation factor XIIIa and the mast cell protease 4 (Figure 2B). The degradomes of ADAMTS2 and ADAMTS14 are common at 59% (40/68) when considering specifically the extracellular and cell surface proteins, while only a 40% (148/369) overlap is found when considering all the other proteins, showing an enrichment for common extracellular substrates (Figure 2B). Investigation of the biological processes (pantherdb.org) related to these degradomes clearly illustrated a fundamental role for these two enzymes in collagen fibril organization, which was expected for ADAMTS2 but was more surprising for ADAMTS14 since collagen fibrils in TS14^−/−^ mice appear to be normal. This analysis also shed light on their implication in lipoprotein regulation and assembly, notably by the cleavage of the proapolipoprotein A-II and apolipoprotein A-I, and in immune response by the regulation of cytokines and chemokines secretion and complement activation, notably through the cleavage of complement proteins (C3C, C4-B) and of the macrophage migration inhibitory factor (Figure 2C, Table 1, Supplementary Figure S1).

**TABLE 1:** Potential extracellular substrates specific or common of ADAMTS2 and/or ADAMTS14.

Name	TS2	TS14	TS2TS14	Name	TS2	TS14	TS2TS14
Inter alpha-trypsin inhibitor	1.2 ± 0.7	1.9 ± 0.7	1.6 ± 0.5	Galectin-1	1.3 ± 0.3	1.9 ± 0.4	2.1 ± 0.4
AE-binding protein 1	0.7 ± 0.2	2.0 ± 0.5	1.2 ± 0.3	Galectin-7	2.0 ± 0.6	2.8 ± 0.8	4.8 ± 1.4
Alpha-2-HS-glycoprotein	0.7 ± 0.3	2.2 ± 1.3	0.9 ± 0.3	Gc-globulin	1.7 ± 0.8	4.1 ± 2.0	6.0 ± 3.7
Alpha-2-macroglobulin	1.7 ± 1.0	2.0 ± 0.6	2.2 ± 0.6	Gelsolin	2.6 ± 0.7	2.4 ± 0.9	5.0 ± 1.7
Annexin-8	6.4 ± 2.2	5.5 ± 1.9	15.0 ± nd^a^	H2-Q9	1.9 ± 1.3	2.7 ± 1.1	2.9 ± 1.1
Apolipoprotein A-I	2.7 ± 1.8	3.3 ± 1.9	4.5 ± 1.8	Hemopexin	1.0 ± 0.4	1.9 ± 0.5	1.5 ± 0.5
Apolipoprotein H	1.6 ± 0.8	2.6 ± 1.2	2.3 ± 0.8	Ig gamma-2B	1.8 ± 0.5	2.3 ± 0.8	3.5 ± 1.2
Beta-globin	2.2 ± 0.6	2.9 ± 1.1	6.7 ± 3.3	Ig gamma-3	1.1 ± 0.4	1.9 ± 0.4	1.8 ± 0.4
Biglycan	2.8 ± 2.4	3.0 ± 1.6	3.6 ± 1.8	Ig kappa chain V-V	1.2 ± 0.8	5.1 ± 2.7	2.9 ± 1.1
Carboxypeptidase A3	1.6 ± 0.4	2.1 ± 0.5	3.2 ± 1.1	Kallikrein j	2.0 ± 0.8	2.4 ± 0.6	5.1 ± 0.6
Cathepsin B heavy chain	1.3 ± 0.6	1.9 ± 0.6	1.6 ± 0.3	Kininogen-1	2.2 ± 0.5	2.9 ± 0.6	5.5 ± 0.2
Cathepsin H	1.2 ± 0.6	2.4 ± 0.8	2.0 ± 0.6	Lumican	1.6 ± 0.7	2.6 ± 1.3	2.3 ± 0.4
Cathepsin S	1.0 ± 0.5	2.2 ± 0.7	1.5 ± 0.4	Mast cell protease 4	1.5 ± 0.2	1.7 ± 0.2	2.4 ± 0.5
Caveolin-1	2.0 ± 0.8	3.1 ± 1.4	3.1 ± 0.0	MIF	1.1 ± 0.6	2.7 ± 1.1	1.8 ± 0.5
Cavin-1	2.1 ± 0.4	3.5 ± 0.9	8.7 ± 0.4	Myelin protein zero	1.6 ± 0.7	2.1 ± 1.1	1.7 ± 0.4
Coagulation factor XIIIa	2.5 ± 1.0	3.8 ± 1.5	7.8 ± nd^a^	Osteoglycin	2.3 ± 0.7	2.3 ± 0.9	4.5 ± 1.6
Collagen alpha-1(I) chain	4.2 ± 0.5	1.7 ± 0.2	6.8 ± 1.0	p24 gamma-1	2.1 ± 1.1	3.3 ± 1.7	1.4 ± 1.0
Collagen alpha-1(III) chain	3.7 ± 2.2	2.8 ± 1.3	5.8 ± 2.7	p35/Annexin A1	2.7 ± 1.2	2.7 ± 0.9	7.4 ± 3.7
Collagen alpha-1(IV) chain	1.6 ± 0.6	1.8 ± 0.6	2.2 ± 0.4	p36/Annexin A2	2.7 ± 1.2	2.7 ± 0.9	7.4 ± 3.7
Collagen alpha-1(V) chain	3.9 ± 1.7	4.1 ± 1.6	9.3 ± 0.8	PCPE-1	2.2 ± 0.4	1.9 ± 0.4	3.6 ± 0.5
Collagen alpha-1(VI) chain	2.2 ± 0.8	1.9 ± 0.6	3.4 ± 0.9	PDI A6	1.8 ± 0.7	2.1 ± 0.7	2.9 ± 0.9
Collagen alpha-1 (XIV) chain	3.6 ± 0.7	2.3 ± 0.5	8.2 ± 2.5	Periostin	1.0 ± 0.5	2.2 ± 0.8	1.3 ± 0.4
Collagen alpha-2(I) chain	3.1 ± 0.7	1.6 ± 0.2	4.6 ± 0.6	Proapolipoprotein A-II	3.5 ± 2.5	4.9 ± 3.2	11.9 ± 7.9
Collagen alpha-2(V) chain	1.8 ± 0.3	1.7 ± 0.3	2.8 ± 0.3	Prolargin	2.9 ± 0.9	2.6 ± 0.8	5.8 ± 1.3
Collagen alpha-2(VI) chain	2.5 ± 0.7	2.2 ± 0.6	4.1 ± 0.0	Protein unc-80 homolog	1.8 ± 0.6	2.0 ± 0.2	3.5 ± 1.4
Collagen alpha-3(VI) chain	4.7 ± 1.3	2.1 ± 0.6	8.0 ± 1.2	Serpin A1c	1.1 ± 0.3	2.4 ± 1.0	2.1 ± 0.5
Complement C3	1.8 ± 1.4	4.3 ± 3.4	2.3 ± 0.8	Serpin A1d	0.8 ± 0.5	2.8 ± 1.3	1.0 ± 0.4
Complement C4-B	0.3 ± 0.1	2.7 ± 1.0	0.6 ± 0.2	Serpin B6	4.7 ± 2.5	3.9 ± 2.4	8.0 ± 1.9
Complement factor B	0.9 ± 0.2	2.0 ± 0.4	1.8 ± 0.5	Protein serpinb6e	1.3 ± 0.3	2.3 ± 0.6	2.5 ± 0.1
Complement factor H	1.0 ± 0.3	1.8 ± 0.5	1.7 ± 0.6	Serum albumin	3.2 ± 1.7	4.2 ± 2.4	12.6 ± 9.7
Corneodesmosin	1.5 ± 0.4	1.7 ± 0.4	2.2 ± 0.4	Siderophilin	2.2 ± 0.7	2.8 ± 1.1	5.2 ± 2.5
Cystatin-3	2.4 ± 0.6	2.7 ± 0.8	7.0 ± 3.7	Stromelysin-1/MMP3	0.4 ± 0.1	1.5 ± 0.3	0.5 ± 0.1
Dermatopontin	2.0 ± 0.4	1.6 ± 0.2	3.1 ± 0.6	Susd 4	2.3 ± 0.7	2.4 ± 1.1	3.9 ± 1.1
Dermokine	1.1 ± 0.3	1.4 ± 0.3	1.4 ± 0.2	Transcobalamin II	0.8 ± 0.5	2.2 ± 1.1	0.8 ± 0.2

^a^ nd: not determined.

The 68 extracellular and cell surface potential substrates are reported together with the average P/C ratio from at least two experiments for ADAMTS2, ADAMTS14 or ADAMTS2 and ADAMTS14. When several peptides were identified for a protein, the ratios from the peptide giving the highest value are reported for illustration. All the peptides and ratios are reported in supplemental.

### Proteomic Analysis of Type I Collagen Processing by ADAMTS2 And/or ADAMTS14 in Mouse Skin

#### Cleavage of the Aminopropeptide of Fibrillar Collagens

As a positive control assessing the quality and the specificity of our technical approach, we first focused on the cleavages of the aminopropeptides of type I procollagens, the primary substrates of ADAMTS2. For Col1A1 and Col1A2, the highest P/C ratios were detected at sequences corresponding to the published cleavage sites in the NC2 domain: S_151_._152_Q for Col1A1 and A_85_._86_Q for Col1A2 (Table 2). Peptides corresponding to more upstream sequences have P/C ratios largely below 1, evidencing the near absence of intact N-propeptide in Wt skin (Supplementary Table S2). These data clearly validate the reliability of our experimental setting.

**TABLE 2:** Type I collagen processing in mouse skin.

Collagen α1(I) chain				Collagen α2(I) chain			
Peptide Sequence	TS2	TS14	TS2TS14	Peptide Sequence	TS2	TS14	TS2TS14
*NC2 (A150-P167)*				*NC2 (A75-P96)*			
NFAS(151).(152)QMSYGYDEKSAGVSVPGPMGPSGPR	4.2 ± 0.5	1.7 ± 0.2	6.8 ± 1.0	NFAA(85).(86)QYSDKGVSSGPGPMGLMGPR	3.1 ± 0.7	1.6 ± 0.2	4.6 ± 0.6
FASQ(152).(153)MSYGYDEKSAGVSVPGPMGPSGPR	2.0 ± 0.3	1.5 ± 0.2	2.9 ± 0.4	FAAQ(86).(87)YSDKGVSSGPGPMGLMGPR	2.0 ± 0.3	2.0 ± 0.3	1.5 ± 0.2
QMSY(155).(156)GYDEKSAGVSVPGPMGPSGPR	2.4 ± 1.0	1.6 ± 0.3	3.3 ± 1.1	YSDK(90).(91)GVSSGPGPMGLMGPR	2.9 ± 0.4	1.4 ± 0.2	2.9 ± 0.6
SYGY(157).(158)DEKSAGVSVPGPMGPSGPR	3.0 ± 1.0	1.8 ± 0.3	5.2 ± 2.0	SDKG(91).(92)VSSGPGPMGLMGPR	1.9 ± 0.3	1.4 ± 0.2	2.6 ± 0.3
DEKS(161).(162)AGVSVPGPMGPSGPR	2.0 ± 0.3	1.5 ± 0.2	2.9 ± 0.5	DKGV(92).(93)SSGPGPMGLMGPR	1.9 ± 0.2	1.5 ± 0.1	2.8 ± 0.1
EKSA (162).(163)GVSVPGPMGPSGPR	1.9 ± 0.3	1.4 ± 0.1	2.6 ± 0.4	*Triple helix* (*G97-G1113*)			
KSAG(163).(164)VSVPGPMGPSGPR	3.7 ± 0.3	1.4 ± 0.1	5.1 ± 0.2	SSGP(96).(97)GPMGLMGPR	1.9 ± 0.3	1.5 ± 0.2	2.7 ± 0.5
*Triple helix* (<I>G168-P1181</I>)				GPGP(98).(99)MGLMGPR	2.5 ± 0.8	1.8 ± 0.5	3.9 ± 1.6
VSVP(167).(168)GPMGPSGPR	1.8 ± 0.5	1.6 ± 0.4	2.5 ± 0.6	RGIP(336).(337)GPAGAAGATGAR	2.5 ± 0.7	1.7 ± 0.4	3.6 ± 1.0
VPGP(169).(170)MGPSGPR	1.8 ± 0.5	1.5 ± 0.4	2.6 ± 1.0	RPGP(485).(486)IGPAGPR	2.4 ± 0.7	1.6 ± 0.4	3.5 ± 1.3
TGPP(332).(333)GFPGAVGAKGEAGPQGAR	2.1 ± 0.6	1.7 ± 0.3	3.5 ± 1.1	RGTP(600).(601)GESGAAGPSGPIGSR	2.1 ± 0.6	1.6 ± 0.3	3.2 ± 1.1
PPGF(334).(335)PGAVGAKGEAGPQGAR	2.3 ± 0.8	1.7 ± 0.5	3.5 ± 1.7	GESG(604).(605)AAGPSGPIGSR	2.0 ± 0.2	1.3 ± 0.1	2.5 ± 0.4
PGFP(335).(336)GAVGAKGEAGPQGAR	2.4 ± 0.8	1.9 ± 0.5	4.0 ± 1.3	ESGA(605).(606)AGPSGPIGSR	2.0 ± 0.4	1.4 ± 0.2	2.7 ± 0.5
RGFP(485).(486)GADGVAGPKGPSGER	2.6 ± 1.0	1.7 ± 0.4	3.8 ± 1.5	VGAP (636).(637)GSAGASGPGGLPGER	2.3 ± 0.8	1.7 ± 0.4	3.5 ± 1.2
GFPG(486).(487)ADGVAGPKGPSGER	1.8 ± 0.3	1.4 ± 0.2	2.3 ± 0.2	SGDR(699).(700)GEAGAAGPSGPAGPR	1.9 ± 0.4	1.6 ± 0.3	3.0 ± 0.7
AGAQ(608).(609)GAPGPAGPAGER	2.2 ± 0.4	1.6 ± 0.3	3.1 ± 0.3	GDRG(700).(701)EAGAAGPSGPAGPR	1.9 ± 0.3	1.5 ± 0.2	2.8 ± 0.3
PGPI(842).(843)GNVGAPGPKGPR	2.4 ± 0.3	1.9 ± 0.3	4.3 ± 0.2	DRGE(701).(702)AGAAGPSGPAGPR	2.4 ± 0.6	1.7 ± 0.4	3.8 ± 1.1
PPGP(889).(890)VGKEGGKGPR	2.4 ± 0.6	1.4 ± 0.3	3.1 ± 0.8	GEAG(703).(704)AAGPSGPAGPR	2.0 ± 0.2	1.3 ± 0.1	2.5 ± 0.4
AGSP(935).(936)GTPGPQGIAGQR	2.2 ± 0.6	1.6 ± 0.3	3.1 ± 1.0	EAGA(704).(705)AGPSGPAGPR	2.1 ± 0.4	1.5 ± 0.2	3.0 ± 0.7
PGTP(938).(939)GPQGIAGQR	2.6 ± 0.8	1.6 ± 0.4	3.8 ± 1.3	AGAP(969).(970)GPHGSVGPAGKHGNR	2.2 ± 0.5	1.6 ± 0.3	3.4 ± 0.7
KNGD(1,054).(1,055)RGETGPAGPAGPIGPAGAR	2.1 ± 0.5	2.0 ± 0.4	3.6 ± 0.1	RGEP(987).(988)GPAGSVGPVGAVGPR	2.5 ± 0.8	1.6 ± 0.4	3.7 ± 1.4
NGDR(1,055).(1,056)GETGPAGPAGPIGPAGAR	2.0 ± 0.4	1.4 ± 0.2	2.6 ± 0.5	EPGP(989).(990)AGSVGPVGAVGPR	2.2 ± 0.6	1.5 ± 0.3	3.2 ± 1.1
RGET (1,058).(1,059)GPAGPAGPIGPAGAR	1.7 ± 0.2	1.2 ± 0.1	2.1 ± 0.1	PGPA(990).(991)GSVGPVGAVGPR	1.9 ± 0.2	1.3 ± 0.1	2.4 ± 0.4
ETGP(1,060).(1,061)AGPAGPIGPAGAR	2.4 ± 0.7	1.6 ± 0.4	3.5 ± 1.1	AGSV(993).(994)GPVGAVGPR	2.3 ± 0.5	1.5 ± 0.2	3.3 ± 0.9
PAGP(1,063).(1,064)AGPIGPAGAR	2.6 ± 0.8	1.5 ± 0.4	3.7 ± 1.5	SVGP(995).(996)VGAVGPR	1.7 ± 0.2	1.6 ± 0.2	2.6 ± 0.3
*NC1* (*S1182-V1453*)				LKGY(1,031).(1,032)SGLQGLPGLAGLHGDQGAPGPVGPAGPR	2.2 ± 0.6	1.9 ± 0.5	3.6 ± 1.1
GYDF(1,187).(1,188)SFLPQPPQEKSQDGGR	4.4 ± 0.4	9.5 ± 3.6	24.3 ± 7.6	YSGL(1,034).(1,035)QGLPGLAGLHGDQGAPGPVGPAGPR	2.6 ± 1.2	1.7 ± 0.5	3.1 ± 1.0
DTTL(1,224).(1,225)KSLSQQIENIR	2.5 ± 0.9	2.0 ± 0.9	3.4 ± 0.8	GLPG (1,039).(1,040)LAGLHGDQGAPGPVGPAGPR	2.9 ± 0.6	1.9 ± 0.5	5.6 ± 2.4
LKSL(1,227).(1,228)SQQIENIR	2.3 ± 0.6	1.7 ± 0.5	3.2 ± 0.8	LPGL(1,040).(1,041)AGLHGDQGAPGPVGPAGPR	2.3 ± 0.4	1.3 ± 0.2	2.8 ± 0.3
				PGLA(1,041).(1,042)GLHGDQGAPGPVGPAGPR	2.8 ± 1.2	2.5 ± 1.2	6.1 ± 2.9
				LAGL(1,043).(1,044)HGDQGAPGPVGPAGPR	2.0 ± 0.3	1.6 ± 0.2	2.9 ± 0.5
				GLHG(1,045).(1,046)DQGAPGPVGPAGPR	1.5 ± 0.4	1.8 ± 0.2	2.7 ± 0.9
				LHGD(1,046).(1,047)QGAPGPVGPAGPR	2.2 ± 0.3	1.5 ± 0.2	3.2 ± 0.1
				HGDQ(1,047).(1,048)GAPGPVGPAGPR	1.8 ± 0.2	1.3 ± 0.1	2.4 ± 0.4
				GDQG(1,048).(1,049)APGPVGPAGPR	2.0 ± 0.2	1.4 ± 0.2	2.7 ± 0.5
				RSGQ(1,076).(1,077)PGPVGPAGVR	2.2 ± 0.3	1.5 ± 0.2	3.2 ± 0.1
				*NC1* (*Y1114-K1372*)			
				DATL(1,145).(1,146)KSLNNQIETLLTPEGSR	3.9 ± 3.4	6.0 ± 5.6	3.2 ± 1.0
				LKSL(1,148).(1,149)NNQIETLLTPEGSR	1.6 ± 0.4	2.0 ± 0.6	2.7 ± 0.8
				RLPF(1,347).(1,348)LDIAPLDIGGADQEFR	3.0 ± 0.7	2.0 ± 0.6	5.3 ± 1.2

Cleavage sites observed by proteomics of ADAMTS2 (TS2) and ADAMTS14 (TS14) within α1 and α2 chains of type I collagen. Each cleavage site has been observed at least in two experiments. The sequences of the cleavage sites are reported in bracket according to the UniprotKB numbering. The classical N-propeptide cleavage sites are highlighted in grey.

**FIGURE 3:**
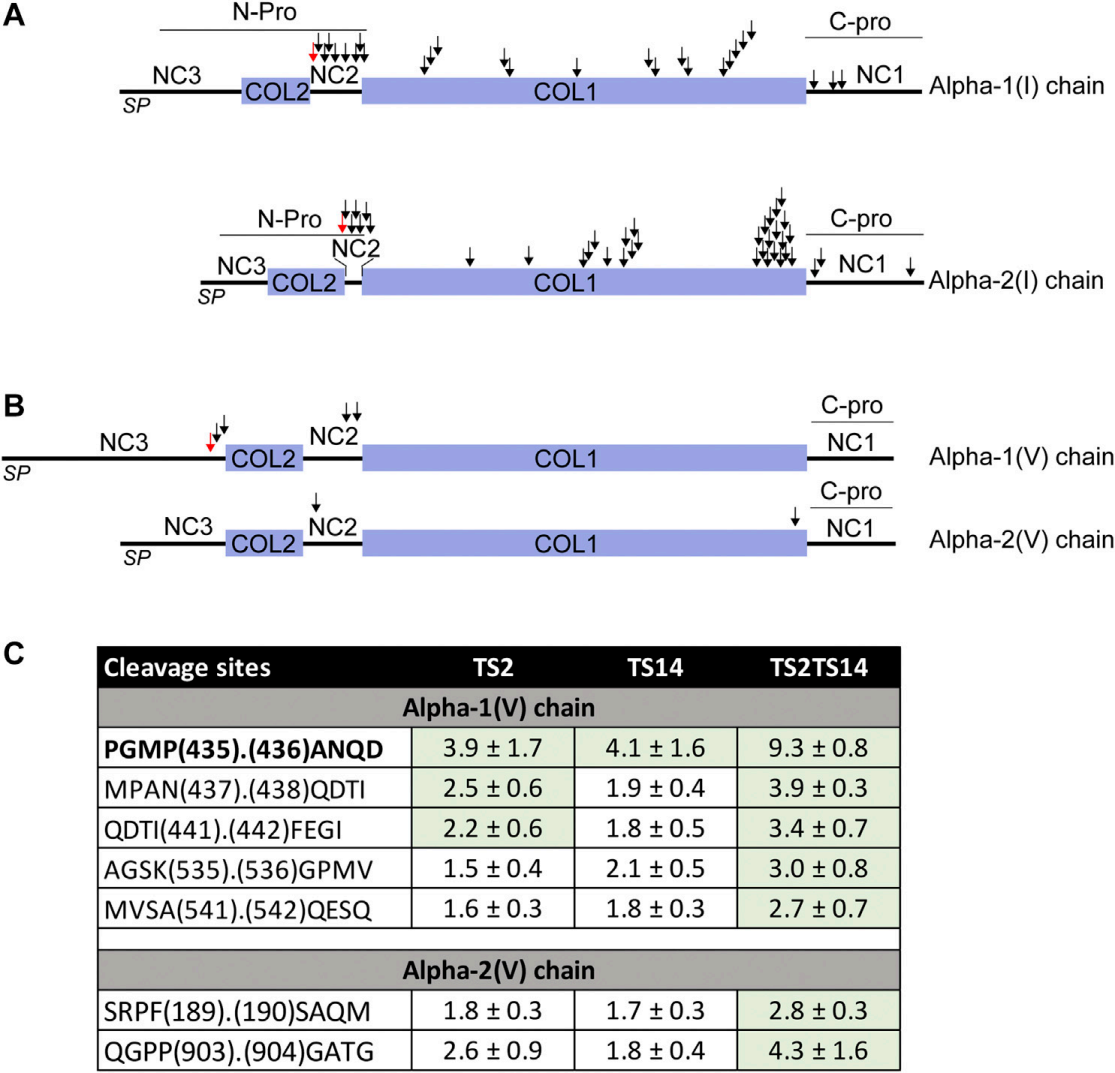
ADAMTS2 and/or ADAMTS14 cleavages of type I and V collagens. Schematic view of the pro alpha-1(I) and alpha-2(I) chains showing the signal peptides (PS), the main triple helix collagenous domain (COL1) and the short triple helix collagenous domain (COL2) and the N- and C-propeptides (N- and C-Pro) **(A)**. Schematic view of the pro alpha-1(V) and alpha-2(V) chains showing the collagenous (COL) and non collagenous (NC) domains **(B)**. The ADAMTS2 and ADAMTS14 cleavage sites are indicated by arrows. The red arrows correspond to the already reported ADAMTS2 cleavage sites (Colige et al., 2005; Bekhouche and Colige, 2015). Table showing the P/C ratio for type V collagens, according to the cleavage sites observed by proteomic. Ratios above the average 3σ cut-offs are highlighted in green. The previously described ADAMTS2 cleavage site within alpha-1(V) chain is in bold **(C)**.

Surprisingly, several additional potential cleavage sites were also identified in the NC2 domain (Figure 3A; Table 2), some with a P/C ratio >2. Some were a few amino acids downstream the “canonical” cleavage site and could therefore result from exopeptidase activity, but several others were at longer distance suggesting that ADAMTS2 cleaves within a preferred region and not exclusively at the previously reported single cleavage site.

Regarding ADAMTS14, no clear activity could be evidenced at sites cleaved by ADAMTS2, which is in line with previous studies (Dupont et al., 2018) showing that type I collagen is fully processed in the TS14^−/−^ mice, and further confirming the specificity of our analyses. Most interestingly, the P/C ratios were, however, higher in TS2^−/−^TS14^−/−^ than in TS2^−/−^ mice suggesting that, in the absence of ADAMTS2, ADAMTS14 can display some aminoprocollagen peptidase activity *in vivo* (Table 2).

The processing of the aminopropeptide of type V procollagens is still a matter of controversy, especially *in vivo*. Processing of Col5A1 has been reported to be performed by BMP1 (within the NC3 domain) and by ADAMTS2 (within the NC3 domain upstream of the Col2 domain) (Figure 3B). However, no cleavage has been reported in the NC2 domains of Col5A1 or Col5A2, while their sequence and localization between two collagen domains are similar to those of type I, II and III fibrillar procollagens which are all processed in this region. Several peptides N-terminally iTRAQ labeled were identified in the large N-terminal domain of Col5A1 encompassing the Col2, NC2 and NC3 domains (Figure 3B). As opposed to what was observed for type I procollagens, the P/C ratios were usually similar for ADAMTS2 and ADAMTS14, suggesting that both enzymes can process Col5A1 with the same efficacy (Figure 3C). Accordingly, the P/C ratios were much higher when comparing Wt and TS2^−/−^TS14^−/−^ samples. The main processing site (P_435_._436_A, upstream of the Col2 domain) was identical to that described previously using recombinant proteins (Colige et al., 2005). As for Col1A1 and Col1A2, other cleavage sites were also identified in this region of Col5A1, two with P/C > 2 (N_437_._438_Q and I_441_._442_F) and one with a P/C of 0.4 (Q_438_._439_D) reflecting a preferential cleavage at N_437_._438_Q. The P/C of the other labeled peptides was not affected by the presence or absence of ADAMTS2 and ADAMTS14, which confirms the specificity of the cleavages with a P/C ratio >2.

Most interestingly, two cleavage sites were also identified in the NC2 domain of Col5A1, including one at an A. Q site as in Col1A2 (Figure 3B). Altogether these data indicate that ADAMTS2 and ADAMTS14 can process the N-terminal portion of Col5A1 at three different sites, meaning that Col5A1 is present in the skin under forms (including that generated by BMP1 cleavage) having N-terminal non-collagenous extremity of different size and bulkiness.

N-terminally iTRAQ-labeled peptides corresponding to the NC2 domain of Col5A2 were also detected. Although the P/C ratios were lower than for Col1A1, Col1A2 and Col1A5, it clearly indicates that the N-propeptide of Col5A2 can be processed by ADAMTS2 and ADAMTS14, but probably at a reduced rate.

Similar to our previous study *in vitro*, the cleavage of the N-propeptide of Col3A1 was not seen in this experiment, probably because the corresponding peptide was not isolated and therefore not analyzed during the MS/MS step.

#### Cleavages Outside of the Aminopropeptide

Having confirmed and further documented the processing of the aminopropeptides of fibrillar collagens, we were also intrigued by the presence of potential cleavage sites elsewhere in type I collagen chains. In a previous work performed *in vitro* using human cells (Bekhouche et al., 2016), we identified several peptides corresponding to the Col1 domain and the C-propeptide of Col1A1 and Col1A2 that were possibly generated by ADAMTS2 or ADAMTS14 (Supplementary Table S3). However, we hypothesized that this could be an artifact linked to the *in vitro* conditions, such as incomplete folding of the triple helical domains leading to an increased sensitivity to proteases. Here, dozens of peptides corresponding to the Col1 domain of Col1A1 and Col1A2 were found to be N-terminally iTRAQ labeled. For both Col1A1 and Col1A2, the P/C ratios were, on average, between 1.5 and two when considering ADAMTS2, and between 1.0 and 1.5 for ADAMTS14 (Supplementary Tables S2, S3). However, similarly to what was observed for the processing of the aminopropeptides, these ratios were significantly higher when comparing Wt and TS2^−/−^TS14^−/−^ skin samples, suggesting that ADAMTS2 and ADAMTS14 can cleave at identical sites. Some “hot spot” regions characterized by several contiguous cleavage sites were found, as, for example, at positions 333 to 337 and 827 to 838 of Col1A1 as well as at positions 988 to 996 of Col1A2 (Table 2). They can be generated by direct cleavages operated by ADAMTS2 and 14 in more sensitive domains or, alternatively, by a single cleavage followed by progressive degradation by exopeptidases. In both cases however, it means that the observed individual P/C ratios give an underestimation of the actual cleavage activity in the concerned region.

Finally, peptides corresponding to cleavages in the C-propeptides were also found. As opposed to our observations for Col1 domain, the P/C ratios were similar for both ADAMTS2 and ADAMTS14, again illustrating the specificity in the identification of potential cleavage sites. For Col1A1, one site (F_1187_._1188_S) is located upstream of the C-propeptide cleavage site by BMP1 (A_1207_._1208_D) while the two others are located about 20 amino acids downstream. For Col1A2, three sites were identified, all after the cleavage site for BMP1 (Table 2).

### Confirmation of the Cleavage of Type I Collagen Within the Col1 Domain and the C-Propeptide

**FIGURE 4:**
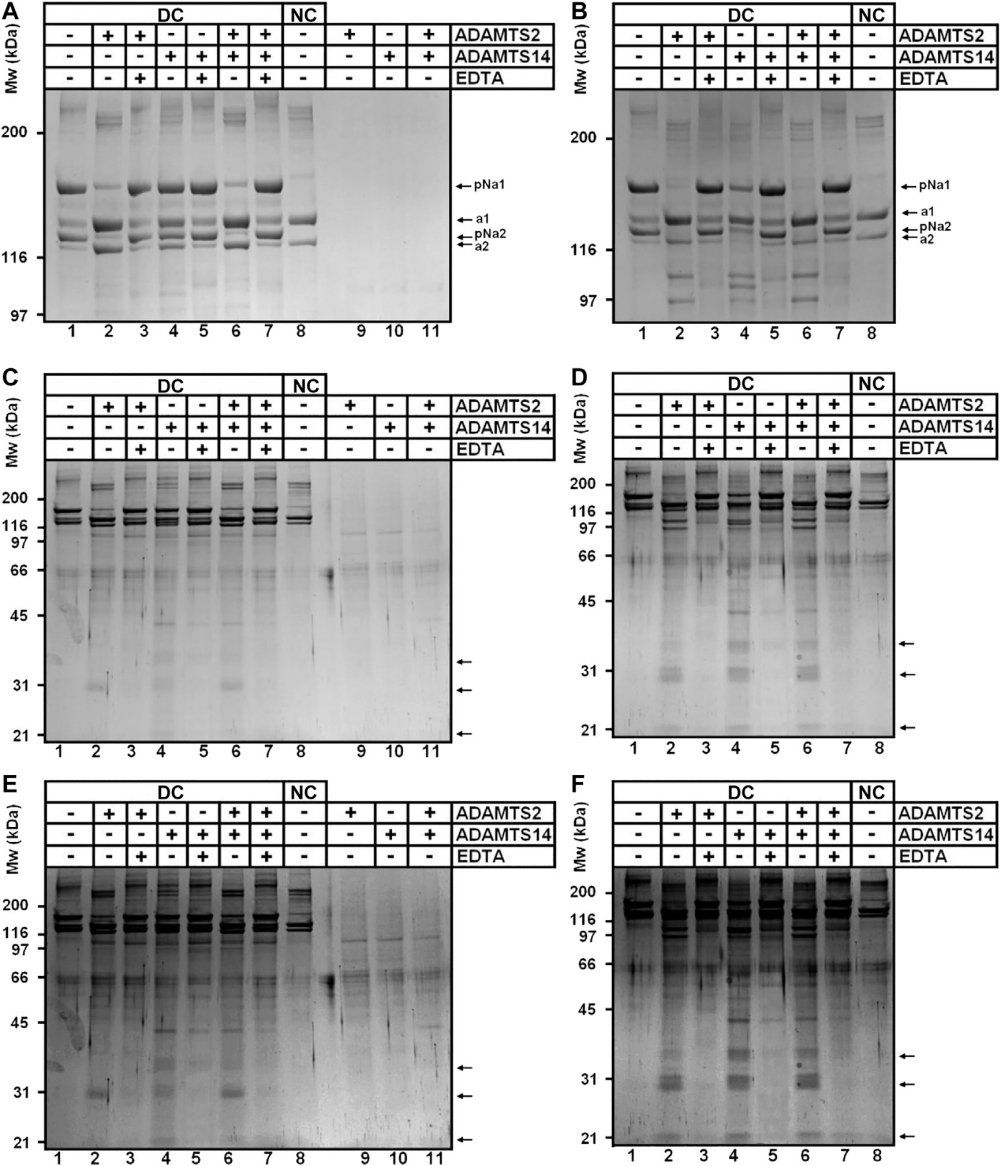
Collagen digestion from calf skin by recombinant human ADAMTS2 and/or ADAMTS14 **(A–F)** Collagen from dermatospatactic calf (DC) skin has been heat denatured (10 min at 95°C) **(B, D, F)** or not **(A, C, E)** before addition of recombinant human TS2 and/or TS14 overnight at 37°C. Digestion products have been analyzed by 6.25% **(A, B)** or 10% **(C–F)** SDS-PAGE stained by Coomassie blue. Pictures at **(E)** and **(F)** correspond respectively to **(C)** and **(D)** with a higher contrast to emphasize the degradation trail. Collagen from normal calf skin (NC) have been used as control for the identification of the fully processed α1 and α2 chains. Products at 31 kDa **(C, E)** correspond to the N-terminal propeptide released mainly by ADAMTS2 when type I collagen is in its native form. Additional low MW products can be observed after incubation of denatured collagen with ADAMTS14 and ADAMTS2.

To confirm our iTRAQ data showing multiple cleavages in the Col1 triple helical domain and in the C-propeptide of type I collagens, we performed *in vitro* assays using recombinant enzymes and collagen purified from the skin of dermatosparactic calf which is characterized by the persistence of the aminopropeptides in about 80% of type I collagen molecules (Figure 4A, lane 1). This particular substrate was chosen because it provides the opportunity to have an internal control consisting in the processing of the aminopropeptide of Col1A1 and Col1A2. This cleavage (conversion of pNa1 and pNa2 into a1 and a2 chains) was almost complete in the presence of recombinant ADAMTS2, but much reduced in the presence of ADAMTS14 in accordance with its lower aminoprocollagen peptidase activity (Figure 4A, lanes 2 and 4, respectively). When looking at lower molecular weight products (Figures 4C,E), the released pNa1 propeptide was observed with an apparent 30 kDa MW and was mainly found in the presence of recombinant active ADAMTS2 (lanes 2 and 6). The presence of other bands and of “trails”, covering the entire migration lanes and corresponding to cleavages of collagen in multiple sites, were also identified (lane 2), including in the presence of recombinant ADAMTS14 (lane 4). Since ADAMTS14 has only a reduced aminoprocollagen peptidase activity, it shows that the two types of activity are not related.

The same type I collagen preparation was also used as substrate after heat denaturation to verify whether disruption of the triple helical folding impacts the sensitivity to cleavage. Processing of the aminopropeptides by ADAMTS2 or ADAMTS14 was still observed, but marked differences were also visible as compared to gels obtained with native collagen (compare panels a, c, e to panels b, d, f of Figure 4), such as the presence of products at 97 and 110 kDa observed in the presence of ADAMTS2 and 14 (lanes 2, 4 and 6 on each panel), and at 105 kDa specifically in the presence of ADAMTS14 (lanes four on each panel). Additional discrete degradation products of lower MW were also identified as well as more pronounced degradation trails along the entire migration lanes (Figure 4, lanes 2, 4, 6; panels d and f).

**FIGURE 5:**
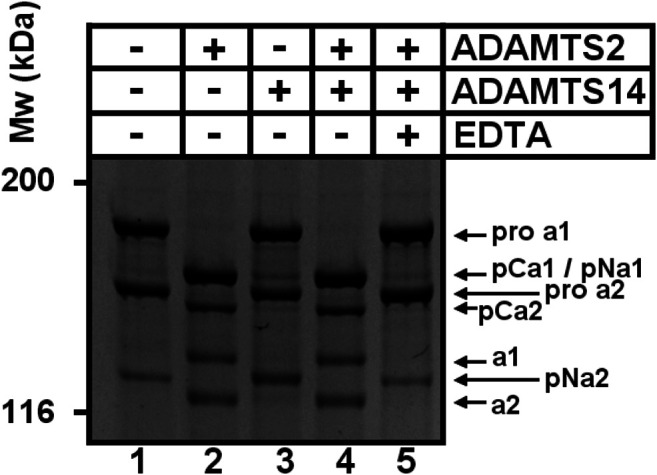
Cleavage of type I procollagen. The collagen substrate, mainly in the form of pro-α1I and pro-α2I, was recovered from culture medium conditioned by dermatosparactic fibroblasts, and then incubated with recombinant human ADAMTS2 and/or ADAMTS14 during 18 h at 37°C. Digestion products were analyzed by 6.25% SDS-PAGE stained by Coomassie blue. The cleavage of pro-α2 into pCα2 and mature α2 demonstrates that ADAMTS2 displays a procollagen C-proteinase activity in addition to its aminoprocollagen peptidase property.

The presence of cleavage sites within the C-propeptide of Col1A1 and Col1A2 was also evidenced by our iTRAQ analyses on *in vitro* assays. Since collagen purified from dermatosparactic skin lacks the C-propeptide, we used collagen produced by human dermatosparactic fibroblasts in culture which is mainly secreted as complete procollagen still retaining its two propeptides (pro-alpha one and pro-alpha 2). After incubation with active ADAMTS2 (Figure 5, lane 2), proa1 and proa2 are converted into pCa1 and pCa2, respectively, as a result of the aminoprocollagen peptidase activity of ADAMTS2. Fully processed alpha1 was also observed while similar amount of pNa1 was absent in the control (lane 1) suggesting that it is produced by cleavage of the N- and C-propeptides of the proa1 chain. In line with this hypothesis, the amount of alpha2 chain recovered after incubation with ADAMTS2 exceeded the amount of pNa2 in the control. Moreover, the relative intensity of pCa2 was lower after incubation with ADAMTS2 (lane 2) than expected from the intensity of proa2 in the control. These two observations clearly suggest some cleavage of the C-propeptide of proa2. Regarding ADAMTS14, only a low aminoprocollagen peptidase activity was observed, as expected, as illustrated by the presence of low amounts of pCa2 generated from proa2 (lane 3). However, accumulation of pNa2 was clearly observed indicating that ADAMTS14 can cleave the C-propeptide of Col1A2 more efficiently than its N-propeptide.

### Other Potential Substrates Involved in Collagen Fibril Formation

Several proteoglycans, non-fibrillar collagens and matricellular proteins are known to be involved in the regulation of collagen fibril formation and functions. Some of them were found to be potential substrates of ADAMTS2 and/or ADAMTS14 based on P/C ratios significantly >2 (Table 1). They are therefore mentioned, although cleavages were not confirmed by other methods, since their processing could be involved in the clinical features found in dermatosparactic EDS and other connective tissue disorders.

Two sites of cleavage in type XIV collagen were found for both ADAMTS2 and ADAMTS14: at positions 630 (AQQY.LEID) within the fifth fibronectin type-III domain and 1,636 (MARY.TAIL) within the third collagen like domain. Identical sites for both enzymes strongly suggest that the cleavages are real and that type XIV collagen is a true substrate. Potential cleavages were also found in COL4A1 (G_1438_._1439_T), at a position corresponding to the release of the arresten cryptic bioactive fragment and in type VI collagens: (1) in the first VWFA domain of COL6A1 (F_186_._187_S); (2) in the VWFA1 and VWFA2 domains of COL6A2 (F_116_._117_S and F_141_._142_A) and (3) in the VWFA6 domain of COL6A3 (F_1051_._1052_A). Besides collagens, potential proteolytic cleavages were also found in PCPE-1 (a co-factor stimulating the processing of the C-propeptide of fibrillar collagens by BMP-1) and in three proteoglycans regulating matrix assembly: biglycan, lumican and osteoglycin. Finally, the degradomes of ADAMTS2 and ADAMTS14 in mouse skin point out several proteins involved in the immune system such as immunoglobulins, complement proteins (C3, C4-B, factor B, factor H), the macrophage inhibitory factor or annexins A8, A1 and A2 known to be involved in leukocyte recruitment and activation (Swisher et al., 2007; Poeter et al., 2014; Sugimoto et al., 2016).

### Potential Intracellular Cleavages

**FIGURE 6:**
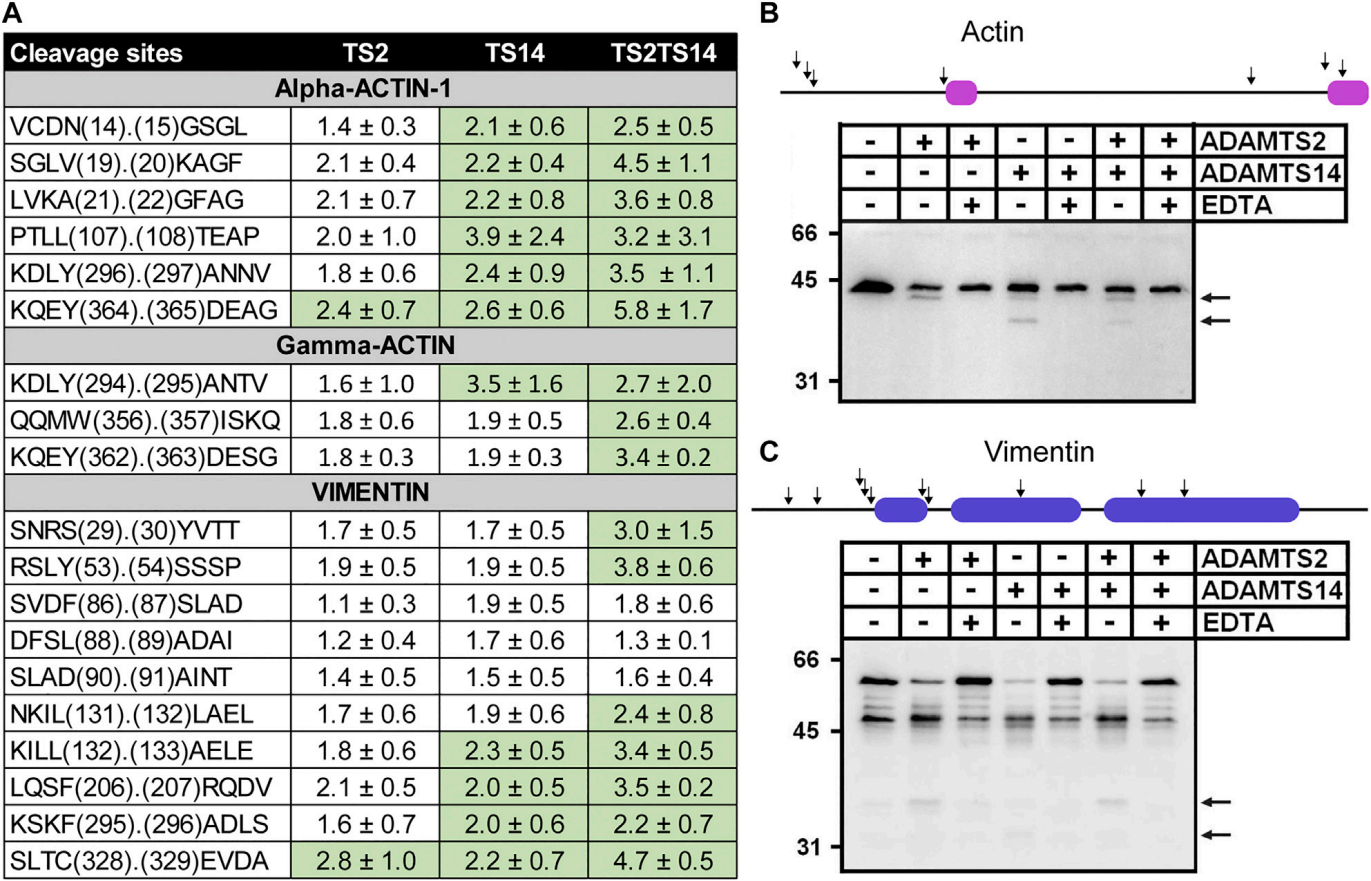
*In vitro* cleavage of actin and vimentin by ADAMTS2 and ADAMTS14. Table showing the P/C ratios according to the cleavage sites observed by proteomic. Ratios above the average 3σ cut-offs are highlighted in green **(A)**. Western blot analysis of *in vitro* cleavage of actin **(B)** and vimentin **(C)** by ADAMTS2 and/or ADAMTS14. The schematic representation of actin and vimentin are reported on the top of the western blots with their respective actinin binding (magenta) and coiled coil (blue) domains. Human fibroblasts lysates were incubated in the presence of recombinant ADAMTS2 and/or ADAMTS14 (200 nM, 16 h at 37°C). EDTA was used as an inhibitor of metalloproteinases. Degradation products are indicated by black arrows.

This work was primarily focused on extracellular and cell surface degradome since ADAMTS2 and ADAMTS14 are secreted proteases. Unexpectedly however, numerous cleavage sites related to the presence/absence of ADAMTS2 and/or ADAMTS14 were found in intracellular proteins (Supplementary Table S1), including actins and vimentin (Figure 6A). On average, P/C ratios for actin were higher for ADAMTS14 than for ADAMTS2, as opposed to what was seen for the N-propeptides cleavages, and even higher when comparing Wt skin and TS2^−/−^TS14^−/−^ skins. Of note also, cleavage sites in actin-2, also known as gamma-actin, are identical to the corresponding cleavage sites in actin-1 which suggests their specificity. In order to confirm these surprising observations, fibroblasts extracts were incubated in the presence of purified ADAMTS2 and/or ADAMTS14 with or without a saturating concentration of EDTA used as inhibitor (Figure 6B). In the absence of ADAMTS and EDTA, actin was detected as a single band of 42 kDa with an antibody raised against the C-terminal decapeptide of alpha-actin-1, demonstrating absence of cleavage by intracellular endogenous proteases, while a second product of about 40 kDa was observed after incubation with ADAMTS2, which would correspond to cleavages at positions V_19_._20_K and/or A_21_._22_G in alpha-actin-1 (Figure 6A). This product was also seen with ADAMTS14, in addition to a second one at about 34–35 kDa which was not observed with ADAMTS2. Remarkably, only discrete bands were seen, with no trace of smear which would indicate multiple cleavages at specific positions.

Regarding vimentin, several peptides with N-terminal iTRAQ labeling were detected, some with P/C ratio slightly but significantly higher in the presence of ADAMTS2 or ADAMTS14 (Figure 6A). For confirmatory purposes Western blot analysis was performed on fibroblast extracts incubated or not with purified ADAMTS2 or ADAMTS14. In the control samples, vimentin appeared as two major bands at 48 kDa and at 58 kDa the latter likely corresponding to the full-length protein. Additional minor products at 53, 50, 46 and 39 kDa were also visible (Romano et al., 2020) (Figure 6C). Upon incubation with ADAMTS2 or 14, the intensity of bands at 58 and 53 kDa were markedly reduced, accompanied by an increased intensity of the products at 48 and 39 kDa and the presence of additional bands at 37 and 32 kDa. These data clearly suggest the existence of several cleavage sites by ADAMTS2 and ADAMTS14 but were not investigated further.

### Cleavage Site of ADAMTS2 and ADAMTS14 in Mouse Skin

**FIGURE 7:**
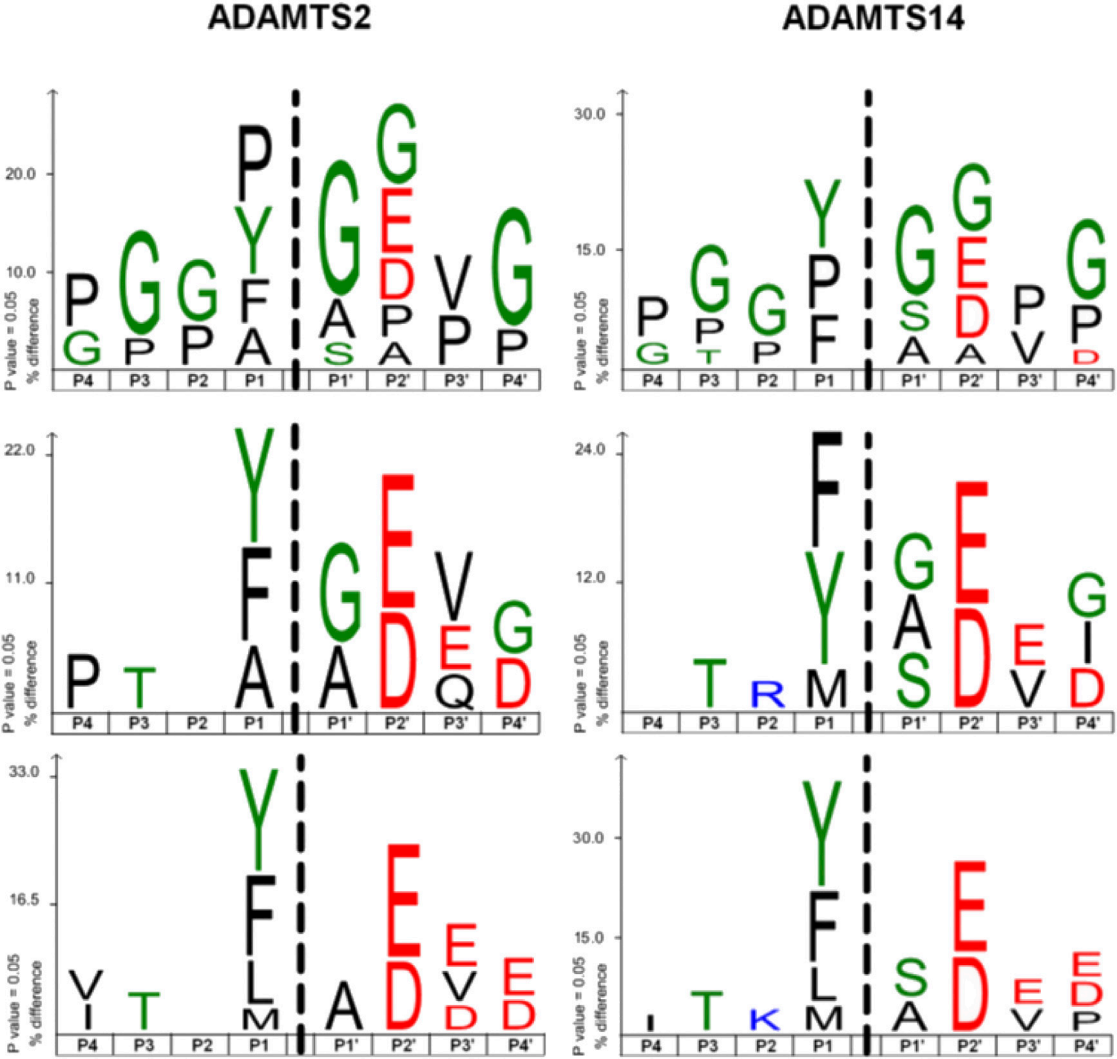
Cleavage site specificity of ADAMTS2 and ADAMTS14 in mouse skin. The cleavage site specificity of ADAMTS2 **(left panels)** or ADAMTS14 **(right panels)** was determined using all the extracellular or extracellular transmembrane cleavage sites **(top)**, without type I collagen **(middle panels)** or without all the fibrillar collagens **(down panels)**. The amino acid sequence logos, corrected by the natural abundance of amino acids in the human proteome, were generated using the iceLogo software package (Colaert et al., 2009). The height of the amino acids represents their frequency regarding their natural abundance; the color reflects its physicochemical properties.

The cleavage sites observed by N-terminomics were used to evaluate cleavage sites enrichment linked to the presence of ADAMTS2 and ADAMTS14. All the potential cleavage sites were used, in a first step, to establish the privileged consensus cleavage sites (Figure 7, upper panel). This revealed an overrepresentation of G and P that could be linked, at least in part, to the abundance of these amino acids in collagens. In addition, Y, F and A were often found at P1 and A and S at P1’, which is similar to previously described cleavage sites for aminoprocollagen peptidases (Bekhouche et al., 2016; Janssen et al., 2016) (Figure 7, upper panel). Considering all the potential extracellular substrates, excluding type I collagen (middle panel) or all fibrillar collagens (lower panel), it was shown that ADAMTS2 and ADAMTS14 display common preferential cleavage sites, enriched in small nonpolar, amphipatic or slightly hydrophobic amino acids (G, P, F, Y, A, L and M). Of note also was the presence of acidic amino acids at P2’, P3’ and P4’.

## Discussion

The procollagen N-proteinases were originally described only for their ability to specifically excise the N-terminal propeptide of fibrillar collagens (Bekhouche and Colige, 2015). However, more recently, we and others have demonstrated their roles in TGFβ signaling (Bekhouche et al., 2016) and in lymphangiogenesis through the proteolytic activation of pro-VEGF-C into VEGF-C able to interact with its receptors (Janssen et al., 2016; Brouillard et al., 2017; Dupont et al., 2018; Wang et al., 2020). The existence of marked differences between the phenotype of patients with arthrochalasia EDS (caused by the absence of the ADAMTS2 cleavage sites in type I collagen) and the phenotype of patients with dermatosparactic EDS (null mutations in ADAMTS2) is another observation arguing for additional functions of ADAMTS2. Finally, the peculiar atopic dermatitis-like skin phenotype of mice deficient in both ADAMTS2 and ADAMTS14 (Dupont et al., 2018) also points to yet to be discovered new substrates of these two related enzymes. In order to search for such potential substrates using a large-scale unbiased approach, we have compared N-terminomes of the skin of Wild type, TS2^−/−^, TS14^−/−^ and TS2^−/−^TS14^−/−^ mice, an experimental approach never tried before because its inherent difficulties, such as the dynamic nature of the cleavage events that could be hidden by cellular uptake, rapid degradation of the generated peptides, and potential variations in protein composition or cell abundance. Moreover, the deficiency of a protease *in vivo* can be compensated by another protease cleaving near or at the same cleavage site (Fortelny et al., 2014). As an additional challenge, skin is formed by several compartments such as *epidermis*, dermis, and muscular, nerve and blood vessels elements which may vary in abundance, a situation susceptible to induce modifications in the relative abundance of a specific protein. The use of a high throughput method and the complexity of the skin samples led to variations between the three individual experiments which prevented the identification of potential gender differences. In this study, three individual experiments have therefore been analyzed independently, in order to increase the likelihood of identifying new substrates, and have been compared between each other in a second step for validations purposes. Potential substrates were identified by taking into account only peptides harboring a P/C ratio above or below the 3-sigma cutoff from the normal distribution of natural N-termini, which constitutes a very stringent threshold. Sixty-eight proteins located at the cell surface or being part of the extracellular matrix were identified as potential substrates of ADAMTS2 and/or ADAMTS14, from which fibrillary collagens were the most represented in terms of identified peptides.

The previously described cleavage sites by ADAMTS2 of the N-propeptide of COL1A1 and COL1A2 were clearly identified, confirming the relevance of our experimental model. Of notes, however, these cleavages seem to occur within a preferential sequence rather than at a specific cleavage site as always reported so far, which illustrates that ADAMTS2 is capable to cleave multiple peptide bonds. As an additional evidence of the specificity of the N-TAILS data, cleavages of the N-propeptide of COL1A1 and COL1A2 by ADAMTS14 were only marginal, which was expected from previous *in vitro* and *in vivo* data showing that ADAMTS14 displays only a very low aminoprocollagen peptidase activity (Colige et al., 2002; Dupont et al., 2018). Having clearly demonstrated the reliability of our experimental design, we then investigated the cleavages of the N-propeptides of type V procollagens which are still incompletely defined. For COL5A1, a cleavage by BMP1 was previously identified within the NC3 domain (Imamura et al., 1998; Bonod-Bidaud et al., 2007) but could not be identified here as expected when using models comparing Wt to Adamts2 and/or Adamts14 deficient skins. The P_435_._436_A site previously found for ADAMTS2 at the end of the variable domain (Colige et al., 2005) was identified here for Adamts2 but also for Adamts14. Accordingly, the P/C ratio was much higher by comparing Wt skin to TS2^−/−^TS14^−/−^ skin, indicating that both enzymes display this activity *in vivo* and, therefore, identifying a new function for ADAMTS14. Some cleavages by both enzymes were also identified in the NC2 domain of COL5A1 and COL5A2 separating the small and the large central triple helical domains, at a location similar to the ones reported for type I, type II and type III procollagens, which was never described before. The existence of several cleavage sites generating type V collagens with N-propeptides of different size and bulkiness is a new observation and is probably part of the regulation operated by type V collagen on collagen fibril formation (Linsenmayer et al., 1993; Marchant et al., 1996; Wenstrup et al., 2004) as observed *in vitro* and *in vivo* in EDS. It would also explain why arthrochalasic and dermatosparactic EDS have different clinical manifestations since both type I and type V collagen processing are altered in dermatosparaxis while only type I collagen is affected in arthrochalasia.

Besides cleavages of the N-propeptides, N-TAILS identified also several cleavages in the central COL1 domain of both alpha one and alpha 2 type I collagens. While some sites were not, or only barely, affected by the presence of ADAMTS2 or 14 (P/C < 1.5), some others were characterized by higher ratios, especially in some clusters, and when comparing Wt to TS2^−/−^TS14^−/−^ skin, which suggested specificity in these cleavages. This hypothesis was evaluated *in vitro* using purified enzymes and purified collagen, either native or heat denatured. It showed that ADAMTS2 and ADAMTS14 can cleave within the COL1 triple helical domain, but mainly (or only) when collagen is unfolded. This “collagenase” activity was similar for ADAMTS2 and ADAMTS14, while ADAMTS14 displays only a limited capacity to cleave the aminopropeptide of non-denatured procollagen which demonstrates that the two activities are independent. The existence of such function was never described before and its biological relevance will have to be further investigated. An attractive hypothesis would be that, when ADAMTS2 (or ADAMTS14) meets and interacts with its type I procollagen substrate, it cleaves the N-propeptide but it also degrades collagen trimers that are not correctly folded, therefore preventing their integration in fibrils. This quality control function of aminoprocollagen peptidases, if confirmed, would also explain why collagen fibrils have a so irregular and abnormal shape in dermatosparaxis. Indeed, in the absence of ADAMTS2, aminoprocollagen but also collagen with defects in the triple helix domains can both accumulate in fibrils and hamper the highly organized polymerization process of collagen fibrils.

Excision of the C-propeptide of type I procollagen was originally attributed to BMP1, and later extended to meprins α and β (Kessler et al., 1996; Jefferson et al., 2013). Here, our degradomic analysis has revealed major cleavage sites for ADAMTS2 and ADAMTS14 in the C-propeptide of both COL1A1 and COL1A2. This was also confirmed *in vitro* using purified enzymes and type I procollagen, and is reminiscent to what was already demonstrated for type III procollagen (Bekhouche et al., 2016). However, the functional relevance of these observations *in vivo*, when BMP1 is present and active, is not clear yet since, except for one site in COL1A1, they are located downstream of the BMP1 site. In these conditions, these ADAMTS-dependent cleavages should not affect the maturation of type I collagen and its polymerization. Since the C-propeptide of type I collagen can inhibit collagen synthesis when released in the extracellular space (Mizuno et al., 2000), an intriguing hypothesis would be that its cleavage by ADAMTS2 or 14 would affect its regulatory function.

The main purpose of this study was to get a better insight into the overall implications of ADAMTS2 and ADAMTS14 in collagen fibril homeostasis. In addition of cleavages at multiple sites in fibrillary collagens, other proteins regulating collagen fibrillogenesis were also found to be potential substrates, similarly to what was found previously regarding the cleavage of Lox (Rosell-García et al., 2019), a crucial enzyme for the formation of crosslinks stabilizing collagen fibrils. Two cleavages were found in COL14A1, a FACIT (Fibril Associated Collagen with Interrupted Triple helix) known for its capacity to interact with collagen fibrils and regulate their formation (Young et al., 2002; Agarwal et al., 2012). Similarly, type VI collagen was also identified as a likely substrate regulating fibril formation (Lamandé and Bateman, 2018; Wu and Ge, 2019). However, the functional relevance of these cleavages was not investigated further but would deserve additional characterizations.

Lumican, biglycan and osteoglycin are members of the Small Leucin-rich Proteoglycan (SLRP) family (Frikeche et al., 2016). They are considered as collagen fibril-regulating proteins, and are also emerging as factors controlling immune response. It is interesting to note that these three proteoglycans have been identified here as potential substrates of ADAMTS2 which is also implicated in immune response as previously described (Hofer et al., 2008) and further illustrated here by the specific enrichment in biological processes linked to complement activation and regulation of cytokines involved in immune response. Although still to be considered as a working hypothesis and having to be confirmed by complementary approaches, these observations could pave the way to better understand the multiple roles of ADAMTS2 and 14 in extracellular matrix formation and functions.

Analysis of the skin degradome also identified cytoplasmic proteins, such as actin and vimentin, as potential substrates of ADAMTS2 and 14. These surprising data were confirmed *in vitro* using skin fibroblasts extracts incubated with recombinant purified enzymes, but not by comparing the electrophoretic pattern of actin and vimentin in Wt and ADAMTS2-deficient fibroblasts. This could suggest that these cleavages occur only after secretion or release in the extracellular space, as observed after cell death for example. We cannot however completely rule out the possibility that ADAMTS2 and 14 could display intracellular activities, either during the secretion process, as described for procollagen processing, or after their internalization when bound to cell membrane (Dubail et al., 2010). The proteomic identification of intracellular substrates has already been reported for MMP2(auf dem Keller et al., 2013) and by studies reporting the intracellular functions of MMPs(Jobin et al., 2017), such as α-actinin cleavage, or the transcriptional regulation of NF-kappa-B inhibitor alpha by MMP12(Marchant et al., 2014). Interestingly, these intracellular activities are related to the innate immunity system (Marchant et al., 2014) with a propensity for MMPs to regulate negatively the pro-inflammatory response (Dufour and Overall, 2013; Khokha et al., 2013). Further studies are needed to precisely assess the role the cleavage of proteins by ADAMTS2 and ADAMTS14 either in the extracellular and/or in the intracellular space, with a potential implication in cytoskeleton dynamic, gene expression and inflammatory response.

## Conclusion

Our N-TAILS analysis of mouse skin degradomes has extended our knowledge regarding the roles of ADAMTS2 and 14 well beyond the previously known cleavage of the N-propeptide of type I, type II and type III procollagens. The identification *in vivo* of several cleavage sites in the N-terminal region of type V collagen, generating N-terminal propeptides of different size and bulkiness, is a new finding possibly explaining how type V collagen can finely tune collagen fibril formation and structure. In the same context, other molecules involved in fibrillogenesis, such as SLRPs and non-fibrillar collagens, have also been identified as likely substrates of ADAMTS2 and 14.

Another intriguing observation is the potential quality control activity leading to the degradation of incorrectly folded collagen trimers before their assembly in collagen fibrils. If this hypothesis is confirmed, this would represent a key activity of ADAMTS2 and 14 for maintaining the structural integrity of collagen fibers and connective tissues.

## Data Availability

The datasets presented in this study can be found in online repositories. The names of the repository/repositories and accession number(s) can be found in the article.
