# Supplementary material for: In vivo N-Terminomics Highlights Novel Functions of ADAMTS2 and ADAMTS14 in Skin Collagen Matrix Building
Source: Front Mol Biosci. 2021 Mar 19;8:643178. doi: 10.3389/fmolb.2021.643178 (PMC8017238; doi:10.3389/fmolb.2021.643178)
Supplement: Supplementary file 1 [file datasheet1.pdf]

Supplemental Figure 1

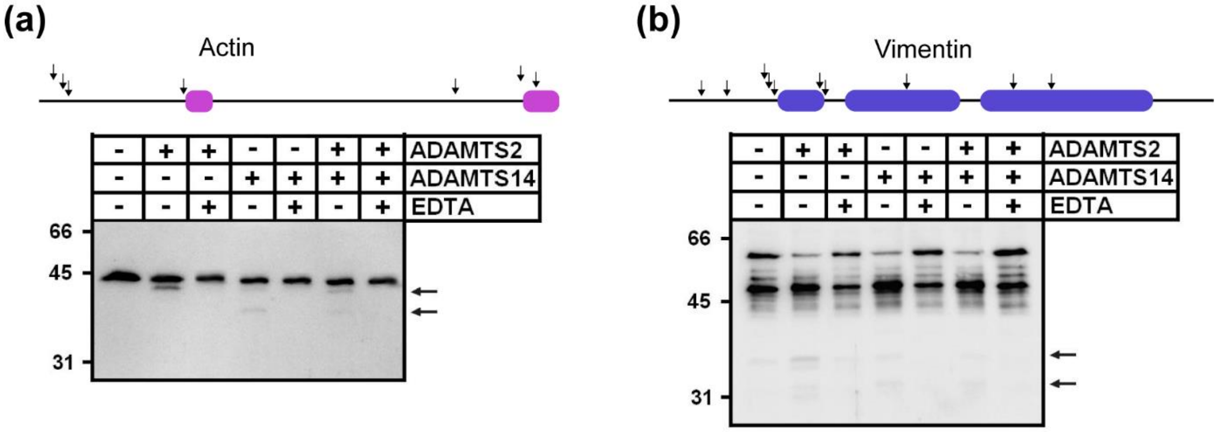

Cleavage of Actin and vimentin from dermatosparactic fibroblasts by ADAMTS2 and ADAMTS14

Western blot analysis of *in vitro* cleavage of actin (a) and vimentin (b) by ADAMTS2 and/or ADAMTS14. The schematic representation of actin and vimentin are reported on top of the western blots with their respective actinin binding (magenta) and coiled coil (blue) domains. Human dermatosparactic fibroblasts lysates were incubated in presence of recombinant ADAMTS2 and/or ADAMTS14 (200 nM, 16h at 37°C). EDTA was used as an inhibitor of metalloproteinases. Degradation products indicated by black arrows are seen after addition of ADAMTS2 and/or ADAMTS14, but not in the absence of enzyme nor in the presence of EDTA which shows the specificity of the cleavages.
